# Understanding Health Care Students’ Perceptions, Beliefs, and Attitudes Toward AI-Powered Language Models: Cross-Sectional Study

**DOI:** 10.2196/51757

**Published:** 2024-08-13

**Authors:** Ivan Cherrez-Ojeda, Juan C Gallardo-Bastidas, Karla Robles-Velasco, María F Osorio, Eleonor Maria Velez Leon, Manuel Leon Velastegui, Patrícia Pauletto, F C Aguilar-Díaz, Aldo Squassi, Susana Patricia González Eras, Erita Cordero Carrasco, Karol Leonor Chavez Gonzalez, Juan C Calderon, Jean Bousquet, Anna Bedbrook, Marco Faytong-Haro

**Affiliations:** 1 Universidad Espiritu Santo Samborondon Ecuador; 2 Respiralab Research Group Guayaquil Ecuador; 3 School of Dentistry Universidad Católica de Santiago de Guayaquil Guayaquil Ecuador; 4 Facultad de Odontología Universidad Católica de Cuenca Cuenca Ecuador; 5 Universidad Nacional de Chimborazo Riobamba Ecuador; 6 Universidad de Las Américas (UDLA) Quito Ecuador; 7 Departamento Salud Pública, Escuela Nacional de Estudios Superiores Universidad Nacional Autónoma de México Guanajuato Mexico; 8 Universidad de Buenos Aires, Facultad de Odontologìa, Cátedra de Odontología Preventiva y Comunitaria Buenos Aires Argentina; 9 Universidad Nacional de Loja Loja Ecuador; 10 Departamento de cirugía y traumatología bucal y maxilofacial Universidad de Chile Santiago Chile; 11 Universidad Politécnica Salesiana Sede Guayaquil Guayaquil Ecuador; 12 Institute of Allergology, Charité – Universitätsmedizin Berlin, Corporate Member of Freie Universität Berlin and Humboldt-Universität zu Berlin Berlin Germany; 13 Fraunhofer Institute for Translational Medicine and Pharmacology ITMP, Allergology and Immunology Berlin Germany; 14 MASK-air Montpellier France; 15 Universidad Estatal de Milagro Cdla Universitaria “Dr. Rómulo Minchala Murillo” Milagro Ecuador; 16 Ecuadorian Development Research Lab Daule Ecuador

**Keywords:** artificial intelligence, ChatGPT, education, health care, students

## Abstract

**Background:**

ChatGPT was not intended for use in health care, but it has potential benefits that depend on end-user understanding and acceptability, which is where health care students become crucial. There is still a limited amount of research in this area.

**Objective:**

The primary aim of our study was to assess the frequency of ChatGPT use, the perceived level of knowledge, the perceived risks associated with its use, and the ethical issues, as well as attitudes toward the use of ChatGPT in the context of education in the field of health. In addition, we aimed to examine whether there were differences across groups based on demographic variables. The second part of the study aimed to assess the association between the frequency of use, the level of perceived knowledge, the level of risk perception, and the level of perception of ethics as predictive factors for participants’ attitudes toward the use of ChatGPT.

**Methods:**

A cross-sectional survey was conducted from May to June 2023 encompassing students of medicine, nursing, dentistry, nutrition, and laboratory science across the Americas. The study used descriptive analysis, chi-square tests, and ANOVA to assess statistical significance across different categories. The study used several ordinal logistic regression models to analyze the impact of predictive factors (frequency of use, perception of knowledge, perception of risk, and ethics perception scores) on attitude as the dependent variable. The models were adjusted for gender, institution type, major, and country. Stata was used to conduct all the analyses.

**Results:**

Of 2661 health care students, 42.99% (n=1144) were unaware of ChatGPT. The median score of knowledge was “minimal” (median 2.00, IQR 1.00-3.00). Most respondents (median 2.61, IQR 2.11-3.11) regarded ChatGPT as neither ethical nor unethical. Most participants (median 3.89, IQR 3.44-4.34) “somewhat agreed” that ChatGPT (1) benefits health care settings, (2) provides trustworthy data, (3) is a helpful tool for clinical and educational medical information access, and (4) makes the work easier. In total, 70% (7/10) of people used it for homework. As the perceived knowledge of ChatGPT increased, there was a stronger tendency with regard to having a favorable attitude toward ChatGPT. Higher ethical consideration perception ratings increased the likelihood of considering ChatGPT as a source of trustworthy health care information (odds ratio [OR] 1.620, 95% CI 1.498-1.752), beneficial in medical issues (OR 1.495, 95% CI 1.452-1.539), and useful for medical literature (OR 1.494, 95% CI 1.426-1.564; *P*<.001 for all results).

**Conclusions:**

Over 40% of American health care students (1144/2661, 42.99%) were unaware of ChatGPT despite its extensive use in the health field. Our data revealed the positive attitudes toward ChatGPT and the desire to learn more about it. Medical educators must explore how chatbots may be included in undergraduate health care education programs.

## Introduction

### Background

Artificial intelligence (AI) and machine learning technologies have transformed various sectors of contemporary society, including health care [1]. Among these developments, AI-powered large language models (LLMs) such as OpenAI’s ChatGPT have shown significant promise in revolutionizing numerous aspects of health care services [2]. ChatGPT is a variation of OpenAI’s language model that generates humanlike writing in a conversational situation [3].

As of January 2023, the population using ChatGPT exceeded 100 million [4]. While ChatGPT was not originally intended for application in health care settings, it is possible that some of these users comprise students or health care practitioners [5]. Consequently, the insights derived from their interactions with ChatGPT may offer valuable information in patient communication, information management, electronic health records, diagnostics, decision-making assistance, and, potentially, therapeutic interventions [6].

LLMs have shown to be beneficial to health care provision [7]. ChatGPT has demonstrated strong, human-level performance supporting decision-making, data management, and patient education in many specialties, such as internal medicine, surgery, and oncology [8,9]. The upcoming generations of health professionals comprise students who undergo training in conditions with plenty of easily accessible technology resources [10]. Some students may assume roles as directors of health institutes, whereas others may engage in research or work as health care professionals. Nevertheless, it is crucial to recognize that the quality of education received will directly impact the caliber of professionals in the future. Consequently, it is imperative to understand the interests that occupy their thoughts concerning the use of tools such as LLMs. This comprehension is essential in determining how these tools can either enhance or fail to enhance their academic and educational competencies as well as their professional application soon after [11].

### Objectives

In light of this, the primary aim of our study was to assess the frequency of ChatGPT use, the perceived level of knowledge, the perceived risks associated with its use, and the ethical issues, as well as attitudes toward the use of ChatGPT in the context of health care education. The second part of the study aimed to assess the association between the frequency of use, the level of perceived knowledge, the level of risk perception, and the level of perception of ethics as predictive factors for participants’ attitudes toward the use of ChatGPT.

## Methods

### Design

This study used a cross-sectional survey among students of health care–related college programs across the Americas to assess their perceptions, attitude, patterns of use, and further learning regarding ChatGPT. This study was conducted from May to June 2023 across all participating countries.

### Sample Size Calculation

The sample size for this study was calculated using the following formula*: n = (Estimated Design Effect Factor × Np[1 – p])/(d2/Z2_1 – α/2_ × [N – 1] + p × [1 – p])*. Accounting for a population size of 1 million, a hypothetical frequency of 50% with a 5% margin of error, and a confidence level of 99.99%, the calculated sample size was 1512.

### Recruitment

Our study focused on individuals aged >18 years enrolled in diverse health care–related college programs such as medicine, nursing, dentistry, nutrition and dietetics, and medical laboratory science. Through a convenience sampling method, we gathered responses from 2661 participants. We adopted a multifaceted recruitment approach to ensure a varied sample of health care students. We reached out to potential participants through email, student networks, social media, on-campus events, academic institutions, and student associations.

We expanded our sample by including universities across the Americas, specifically in Argentina, Mexico, Colombia, Chile, and Ecuador. By disseminating study links to these institutions, we achieved a diverse representation of health care students from different countries and fields.

### Bias

To minimize potential biases, we adopted a comprehensive recruitment strategy targeting a wide range of universities across the Americas, hence reducing selection bias. Response bias was mitigated by conducting anonymous surveys, encouraging honest responses from the participants. In addition, to limit information bias, the survey questions were designed to be straightforward and used standardized Likert-scale responses.

### Questionnaire

The questionnaire was developed following the recommendations by Passmore et al [12] and Eysenbach [13]. A steering committee composed of 4 experts and heads from 4 specialized centers worldwide reviewed the literature and developed the survey items, which integrated all constructs to be assessed. The first section of the survey gathered the demographics and medical education of the participants. The second section of the survey aimed to assess the students’ perceptions, attitudes, patterns of use, and further learning regarding ChatGPT.

The perception domain was further categorized into *self-perceived knowledge*, *ethics*, and *beliefs of perceived risk* subdomains. The subdomain of self-perceived knowledge was assessed on a 5-point Likert scale ranging from 1 (*no knowledge*) to 5 (*superior knowledge*). The scale of self-perception of knowledge about ChatGPT was recategorized as follows: (1) “No knowledge”—this category included participants who either answered “No” to the question “Have you heard of ChatGPT before?” or selected “No Knowledge” in response to the question “How would you rate your knowledge of ChatGPT and its applications in health care?”; (2) “Minimal knowledge”—participants falling into this category included those who answered with options such as “Minimal” or “Basic knowledge” on the Likert scale; and (3) “Adequate knowledge”—this category encompassed participants who selected options such as “Adequate” or “Superior” knowledge on the Likert scale.

The *ethical perception* subdomain featured 3 items, which respondents were asked to score on a 5-point Likert scale ranging from 1 (*totally unethical*) to 5 (*totally ethical*). The *beliefs of perceived risk* subdomain had 3 items, which respondents were asked to score on a 5-point Likert scale (1 [*strongly disagree*] to 5 [*strongly agree*]). The attitude domain included 5 statements reflecting evaluations and opinions on ChatGPT. On a 5-point Likert scale, respondents were asked to score these statements (1 [*strongly disagree*] to 5 [*strongly agree*]). The domain of further learning consisted of 4 questions inquiring as to whether respondents wanted to learn more about ChatGPT. Respondents were asked to choose the resources or educational materials that they believed would be the most beneficial in learning about ChatGPT and its potential applications in health care. Those who did not want to learn more about ChatGPT were requested to explain their reasons.

In total, 2 questions assessed the “Pattern of Use” domain: one assessing the frequency of use using a 5-point Likert scale ranging from 1 (*less than once a month*) to 5 (*more than once a day*) and one assessing the applications of ChatGPT in health care settings with a choice of 8 alternatives.

The questionnaire is shown in Multimedia Appendix 1. A pilot study was performed by the steering committee with colleagues and a sample of 20 students. After drafting the survey, it was distributed to the study population in May and June 2023. The survey was available in English and Spanish.

### Ethical Considerations

Ethics approval was obtained from the Human Research Ethics Committee from Ecuador with approval HCK-CEISH-2022-006. All participants provided informed consent to take part in the study. They were informed about the purpose of the research, their rights as participants, and the voluntary nature of their participation. We ensured the privacy and confidentiality of participant data throughout the study. The survey responses were anonymized, and no personally identifiable information was collected. No compensation was provided to participants for their involvement in the study. It is important to note that the approval obtained from the Human Research Ethics Committee in Ecuador was deemed sufficient to expand recruitment to all Latin American countries included in the study. This decision was made based on the similarity of ethical standards and regulations across these countries, as well as the collaborative nature of the research conducted within the region.

### Variables

#### Demographic Variables

The demographic variables selected for this study are pivotal for examining the diversity of health care students’ attitudes toward using ChatGPT. They are used in both the descriptive (for sample composition purposes) and regression (as control variables) tables. Each variable is thoughtfully coded to capture the nuanced differences among the survey participants, facilitating a detailed analysis of their responses.

Age was recorded as a continuous variable. This allowed for precise analysis of trends across different age groups, helping identify whether younger students are more adept and receptive to AI technologies such as ChatGPT compared to their older counterparts [14].

Gender was categorized into several groups: male, female, nonbinary or third gender, prefer not to say, and other. This categorization ensured that the study could address and respect the diversity of gender identities. It allowed for an analysis of whether perceptions of ChatGPT vary significantly across different gender groups, which could indicate targeted approaches for technology integration based on gender-specific preferences or concerns [15].

The type of university was divided into public and private. This classification helped investigate whether the institutional context influences students’ familiarity with and attitudes toward ChatGPT. Differences in resources, exposure to technology, and educational priorities between public and private universities might contribute to distinct attitudes observed among the students from these institutions [16].

Region was split into Central America and South America. By distinguishing between these 2 regions, the study could explore regional differences that might affect students’ acceptance and use of AI technologies. Such differences could stem from varying levels of technology integration in health care education, regional cultural attitudes toward technology, and economic factors [17].

The field of study was specified as medicine, nursing, nutrition, dentistry, therapy, psychology, pharmacology, and other. This detailed categorization allowed the study to determine whether students in certain fields are more likely to perceive ChatGPT as a beneficial tool [18]. For instance, fields requiring up-to-date information and quick data retrieval might show higher appreciation for AI assistance compared to fields that are more focused on personal patient interactions [19].

#### Outcome Variables

The outcomes of this study focused on the health care students’ attitudes toward using ChatGPT quantified through a series of statements. These statements were designed to capture various dimensions of the perceived utility and reliability of ChatGPT in health care contexts. Each outcome variable was measured using Likert scales ranging from “strongly disagree” to “strongly agree” in order to have a granular view of respondents’ attitudes and, through detailed statistical analysis, assess trends and influences on these perceptions.

Specifically, the outcomes assessed were (1) “I think that ChatGPT makes my job easier.”—this statement evaluated the perceived practical utility of ChatGPT in simplifying tasks within health care settings; (2) “ChatGPT can be beneficial in health care settings.”—this statement assessed broader benefits, looking at whether students believe ChatGPT can positively impact health care environments; (3) “ChatGPT provides trustworthy health care information or guidance.”—this statement measured trust in the accuracy and reliability of the information provided by ChatGPT; (4) “ChatGPT is a useful tool when I need to search for information on specific medical questions.”—this statement evaluated the usefulness of ChatGPT as a resource for specific, actionable medical inquiries; and (5) “ChatGPT is a useful tool when I need to search for medical literature.”—this outcome explored the utility of ChatGPT in supporting academic and professional research within medical fields.

Focusing on these specific attitudes toward using ChatGPT helps us understand how health care students perceive the integration of AI into their practices. The statements target various dimensions of AI’s role—from enhancing efficiency and providing reliable information to supporting academic research—highlighting areas where ChatGPT could be particularly impactful or face resistance. This nuanced approach not only sheds light on current acceptance levels but also pinpoints areas where further education or system improvements might increase trust in and the utility of AI applications within health care environments.

#### Exposure (Predictor) Variables

##### Overview

In this study, several key predictor variables were used to explore the factors influencing health care students’ attitudes toward using ChatGPT. These predictors included knowledge of ChatGPT, perceptions of risk, ethical considerations, and the frequency of use of ChatGPT. A detailed overview of each predictor is presented in the following sections.

##### Knowledge About ChatGPT

For the regression model, this predictor measured the participants’ self-reported knowledge about ChatGPT, assessing their understanding of its functionalities and potential applications in health care. It was quantified using a 5-point Likert scale ranging from 1 (*no knowledge*) to 5 (*superior knowledge*). The understanding of ChatGPT’s functionalities and potential applications is crucial as it directly influences how students perceive its utility and limitations [20]. Higher levels of knowledge might correlate with more positive attitudes as students are better able to appreciate the benefits and manage the limitations of AI in health care [21].

##### Beliefs of Perceived Risk

This variable is a composite score derived from the median of the agreement on a 5-point scale with three specific statements assessing perceived risks associated with AI: (1) “I think my job could be replaced in the future because of AI,” (2) “In the future, ChatGPT (or some similar technology) will play an even more important role in my job,” and (3) “Using AI like ChatGPT in clinical practice raises ethical concerns.”

Perceptions of risk are vital to consider because they shape how students weigh the advantages against the potential drawbacks of using AI technologies [22]. Concerns about job security, the increasing role of AI in health care, and ethical implications could negatively influence their attitudes toward ChatGPT, making it essential to analyze how these perceptions impact their overall acceptance [23].

##### Ethics

The ethical factors were assessed by calculating the median score based on the replies’ level of agreement, ranging from *totally ethical* to *totally unethical* on a 5-point scale, to the following three statements that address ethical concerns about using AI in health care: (1) “Revising the language of a scientific manuscript?” (2) “Writing text in a scientific manuscript?” (3) “The sole source of information for the clinical practice?”

Ethical considerations are paramount in the adoption of any new technology, especially in sensitive fields such as health care. Evaluating how students perceive the ethical dimensions of using ChatGPT for tasks such as manuscript writing or as a clinical information source can provide insights into the ethical acceptability of AI tools in professional health care practices [24].

##### Frequency of Use

The frequency of use was directly measured by asking participants how often they used ChatGPT, with options on a 5-point Likert scale ranging from 1 (*less than once a month*) to 5 (*more than once a day*). The frequency of use is indicative of both familiarity and dependency on the technology. Regular use of ChatGPT might suggest greater comfort and perceived utility, possibly leading to more favorable attitudes [25]. Conversely, infrequent use might indicate skepticism or perceived inadequacies in the technology’s ability to meet professional needs [26].

### Statistical Analysis

#### Descriptive Analysis

In the descriptive analysis, we examined the demographic information and survey responses of the participants. This part of the analysis comprised 2 main components. First, the demographic characteristics of the participants were assessed and stratified according to the participants’ self-rated knowledge of AI. These categories of knowledge were “No knowledge,” “Minimal Knowledge,” and “Adequate Knowledge.” Demographic variables such as age, gender, type of university (public vs private), region, and major were analyzed across these knowledge strata. Statistical significance for differences across the knowledge categories was tested using a chi-square test for categorical variables and an ANOVA for continuous variables, with a *P* value of <.05 indicating statistical significance.

In the second part of the descriptive analysis, given the ordinal nature of the variables, we assessed the range, median, and IQR of scores for each item in the survey. The survey items were grouped into 3 primary domains: perception, ethics, and attitudes, with the perception domain further divided into 2 subdomains: knowledge and beliefs of perceived risk. In addition, the frequency of use of ChatGPT for various tasks was analyzed. Each item was assessed on a Likert scale ranging from 1 to 5 except for the use tasks, which were reported as percentages. The total median scores for each domain and subdomain were calculated and included in the report. This analysis helped provide a clear picture of the participants’ perceptions, ethical considerations, attitudes, and use habits related to ChatGPT.

#### Regression Analysis

Our analysis of the impact of perception scores on attitude variables involved the use of multiple ordinal logistic regression models. Each model evaluated the attitudes of health care students toward the use of ChatGPT, with individual attitude statements serving as dependent variables. These statements included perceptions of ChatGPT in terms of its ease of use, its utility in health care settings, the trustworthiness of its health information, its usefulness in finding answers to specific medical questions, and its helpfulness in searching for medical literature.

For each attitude statement, three perception subdomains were considered as independent variables: knowledge, beliefs of risk, and ethical considerations. The coefficient, SE, 1-tailed *t* test, and *P* value were all calculated for each perception subdomain under each attitude statement. All models were adjusted for control variables, including gender, whether the institution attended was private or public, the field of study, and the country of the student. All analyses were carried out using Stata (version 18.0; StataCorp).

#### Missing Data

Although our web-based survey, which required complete responses, effectively eliminated the need to handle missing data, the self-selecting nature of web-based surveys could introduce some bias. Participants more comfortable with or having better access to technology might be overrepresented. However, the completeness of the data set ensured the accuracy of our analysis and the robustness of the findings.

#### Sensitivity Analyses

In the analytical procedure, we used a set of 20 ordinal logistic regression models. Importantly, SEs were clustered by country to account for potential intracountry correlations. The proportional odds assumption, pivotal for the conventional interpretation of ordinal logistic regression, was violated in half (10/20, 50%) of these models. This breach was primarily attributed to the coefficient of the main predictor in the affected models.

To address this violation and offer a more fitting statistical representation, we used the partial proportional odds model for instances in which the main predictor was unconstrained. Even after this adjustment, our results suggested that the interpretation did not differ significantly from models in which every coefficient was constrained, even when faced with assumption violations. Due to this slight difference in interpretation, and in the interest of consistency, we chose to present the outcomes of all models using ordinal logit with all constraints.

For further refinement of our analysis, and to account for potential clustering effects, we introduced random-intercept and slope models. In this setup, schools were treated as nested entities within countries. This multilevel modeling approach produced results that differed only minimally from those of our initial models, underscoring the reliability of our findings.

## Results

### Demographic Information

This study included 2661 health care students in total. Most were female (n=1764, 66.29%), in dentistry (n=1466, 55.09%), from South America (n=2442, 91.77%), and from private universities (n=1836, 68.99%), as indicated in Table 1. The average age was 21.65 (SD 3.42) years. Multimedia Appendix 2 provides a full overview of the sample’s demographics.

**Table 1:** Demographic information (N=2661).

Variable		No knowledge (n=1142)	Minimal knowledge (n=578)	Adequate knowledge (n=941)	Total	*P* value	
Age (y), mean (SD)		22.01 (3.41)	21.45 (3.81)	21.34 (3.12)	21.34 (3.12)	<.001	
**Gender, n (%)**							<.001
	Male	277 (31.66)	203 (23.2)	395 (45.14)	875 (32.88)		
	Female	858 (48.61)	371 (21.02)	536 (30.37)	1765 (66.32)		
	Nonbinary or third gender	2 (25)	2 (25)	4 (50)	8 (0.01)		
	Prefer not to say	2 (20)	2 (20)	6 (60)	10 (0.01)		
	Other	3 (100)	0 (0)	0 (0)	3 (0.01)		
**Type of university, n (%)**							<.001
	Public	397 (48.18)	175 (21.24)	252 (30.58)	824 (30.96)		
	Private	745 (40.56)	403 (21.94)	689 (37.51)	1837 (69.04)		
**Region, n (%)**							.004
	Central America	116 (53.21)	43 (19.72)	59 (27.06)	218 (8.2)		
	South America	1026 (42)	535 (21.9)	882 (36.1)	2443 (91.8)		
**Major, n (%)**							<.001
	Medicine	212 (23.85)	223 (25.08)	454 (51.07)	889 (33.4)		
	Nursing	36 (73.47)	4 (8.16)	9 (18.37)	49 (1.84)		
	Nutrition	24 (41.38)	13 (22.41)	21 (36.21)	58 (2.17)		
	Dentistry	777 (53)	286 (19.51)	403 (27.49)	1466 (55.09)		
	Therapy	18 (40.91)	7 (15.91)	19 (43.18)	44 (1.65)		
	Psychology	19 (42.22)	19 (42.22)	7 (15.56)	45 (1.69)		
	Pharmacology	13 (86.67)	1 (6.67)	1 (6.67)	15 (0.56)		
	Other	43 (45.26)	25 (26.32)	27 (28.42)	95 (3.57)		

### Perception of Knowledge, Beliefs of Perceived Risks, and Ethics

Among all participants, 42.92% (1142/2661) did not know about ChatGPT. Male students knew more about ChatGPT than female students (598/875, 68.3% vs 907/1765, 51.39%, *P*<.001). Most of the group of participants who had adequate knowledge of ChatGPT were from South America. With the exception of medicine and therapy students, most health care students were unaware of ChatGPT (Table 1).

Table 2 presents findings from our survey assessing participants across multiple domains related to their perception, attitudes, and use of AI, with a particular focus on ChatGPT. In the “Perception” domain, participants were queried about their knowledge, with scores ranging from 1 to 5. They reported a median score of 2.00, which implies a minimal knowledge of ChatGPT. Delving into beliefs about the perceived risk linked to AI, respondents “somewhat agreed” that using ChatGPT raises potential ethical concerns and that AI will play a more important role in their jobs in the future.

Moving to the “Ethics” domain, participants considered the use of ChatGPT for writing text within a scientific manuscript and using ChatGPT as the sole information source for clinical practice “neither ethical nor unethical.” In terms of “Attitudes” toward ChatGPT, the median score was 4.00 among all statements, showing that most participants “somewhat agreed” with the advantages and utility of ChatGPT in health care contexts.

The “Use” domain had respondents spotlight the frequency with which they engaged with ChatGPT, reporting a median score of 2.00 (once a month) on a scale of 1 to 5, with an IQR of 1.00-3.00. Regarding distinct tasks, most participants used ChatGPT for homework support (1078/1519, 70.97%), research paper writing (637/1519, 41.94%), and medical and health care education (349/1519, 22.98%); for more information, see Multimedia Appendix 3.

**Table 2:** Range, median, and IQR of the scores of the survey domains^a^.

Items				Scores, median (IQR; range)
**Perception**				
	Knowledge			2.00 (1.00-3.00; 1-5)
	**Beliefs of perceived risk**			
		“I think my job could be replaced in the future because of AI.”	3.00 (1.50-4.50; 1-5)	
		“In the future, ChatGPT (or some similar technology) will play an even more important role in my job.”	4.00 (3.50-4.50; 1-5)	
		“Using AI like ChatGPT in clinical practice raises ethical concerns.”	4.00 (3.50-4.50; 1-5)	
		Total median score	3.28 (2.76-3.81; 1-5)	
	**Ethics**			
		Revising the language of a scientific manuscript	2.00 (1.00-3.00; 1-5)	
		Writing text in a scientific manuscript	3.00 (2.50-3.50; 1-5)	
		The sole source of information for clinical practice	3.00 (2.00-4.00; 1-5)	
		Total median score	2.61 (2.11-3.11; 1-5)	
**Attitudes**				
	I think that ChatGPT makes my job easier.			4.00 (3.48-4.52; 1-5)
	ChatGPT can be beneficial in health care settings.			4.00 (3.00-5.00; 1-5)
	ChatGPT provides trustworthy health care information or guidance.			4.00 (3.50-4.50; 1-5)
	ChatGPT is a useful tool when I need to search for information on specific medical questions.			4.00 (3.00-5.00; 1-5)
	ChatGPT is a useful tool when I need to search for medical literature.			4.00 (3.00-5.00; 1-5)
	Total median score			3.89 (3.44-4.34; 1-5)
**Use**				
	Frequency of use			2.00 (1.00-3.00; 1-5)

^a^n=1519, which corresponds to students who were aware of ChatGPT.

### Further Learning Regarding ChatGPT

Of the participants willing to learn more about ChatGPT, 67.98% (1809/2661) wanted to learn about the applications of ChatGPT in particular cases of medical practice, followed by homework support and understanding the benefits and limits of ChatGPT (Table 3). Less than 30% (745/2661, 27.99%) were interested in learning about “data privacy and security measures” and “ethical considerations.” Participants found that the most interesting educational materials for learning more about this topic were research articles and case studies (426/2661, 69.16%), internet-based demonstrations or hands-on experience (1301/2661, 48.91%), workshops or conferences (1211/2661, 45.52%), and webinars or web-based courses (968/2661, 36.37%).

**Table 3:** Further learning domain showing aspects of ChatGPT and its applications in health care that students are more interested in learning about (N=2661).

Aspect	Students, n (%)
Specific use cases in medical practice	1832 (68.85)
Academic homework support	1241 (46.6)
Potential benefits and limitations	1158 (43.51)
Integration with existing health care systems	1078 (40.51)
Data privacy and security measures	755 (28.38)
Ethical considerations	750 (28.2)
Other	37 (1.39)

The main reasons for the 16.49% (439/2661) of participants who did not want to learn more about ChatGPT were lack of time (1234/2661, 46.37%); preference to consult with peers, mentors, and teachers (617/2661, 23.19%); not enough knowledge about these technologies (492/2661, 18.5%); and lack of relevance to their medical specialty (335/2661, 12.59%; Table 4).

**Table 4:** Reasons for lack of interest in learning more about ChatGPT and its potential applications in health care (N=2661).

Reason	Students, n (%)
Lack of time	1233 (46.37)
I prefer to consult with my peers, mentors, and teachers	617 (23.19)
Not enough knowledge of these technologies	492 (18.5)
Lack of relevance to my medical specialty	336 (12.65)
Skepticism about the benefits of AI^a^ in health care	299 (11.24)
Already overwhelmed with existing medical knowledge and skills	249 (9.37)
Difficulty or discomfort using computer technology	155 (5.85)
Other	143 (5.39)

^a^AI: artificial intelligence.

### Association Between Perception (Knowledge, Belief, and Ethics) and Frequency of Use and Attitude

The ordinal logistic regression analysis (Tables 5 and 6) illustrates the relationship between predictors such as knowledge, beliefs about risks, ethics, frequency of use, age, gender, institution type, and professional background and their impact on health care students’ perceptions of ChatGPT’s utility.

An enhanced understanding of ChatGPT consistently showed a positive correlation with more favorable views across all outcomes. For instance, as knowledge increased, the odds of believing that ChatGPT makes one’s job easier went up, with odds ratios (ORs) ranging from 1.259 (95% CI 1.047-1.513) to 1.468 (95% CI 1.289-1.672). This trend persisted across other perceptions, such as ChatGPT’s potential benefits in health care settings and its trustworthiness in providing health care information.

Beliefs about risk followed a distinctive pattern. Those with heightened risk beliefs felt that ChatGPT made their job easier and could play a beneficial role in health care settings, including obtaining information on medical questions and as a tool for searching medical literature, as evidenced by ORs of 2.040 (95% CI 1.765-2.358), 1.106 (95% CI 1.031-1.186), 1.179 (95% CI 1.110-1.255), and 1.138 (95% CI 1.076-1.203), respectively. This finding suggests that recognizing potential risks does not negate belief in the tool’s utility. Ethical considerations played a significant role. Students with higher ethical concerns perceived ChatGPT’s potential in health care more favorably. The ORs for these associations were notable, especially in the context of trustworthiness and specific medical queries (OR 1.620, 95% CI 1.498-1.752).

The frequency of ChatGPT use was a significant determinant. Regular users were more optimistic about its utility, which was evident across all outcomes, such as its benefits in health care (OR 1.540, 95% CI 1.420-1.670) and its efficacy in searching for medical information (OR 1.438, 95% CI 1.311-1.577).

Age influenced perceptions. Older individuals generally had a higher OR across the outcome variables, suggesting a more positive perception of ChatGPT’s utility in their profession. Gender-based analysis revealed that female individuals, compared to male individuals, were generally more likely to believe that ChatGPT can help in their job. However, perceptions varied when it came to broader benefits in health care and other outcomes. Those identifying as nonbinary or third gender or those who preferred not to specify their gender showcased diverse perceptions, sometimes differing from those of both male and female individuals.

Institutional type and major played a role. Individuals from private institutions, compared to their public institution counterparts, had varied perceptions. Students from nursing and nutrition exhibited unique outlooks on ChatGPT, highlighting the influence of professional background on shaping perceptions.

**Table 5:** Estimates from ordinal logistic regression models for the effect of perception scores on attitude variables^a^.

Variables and outcomes	Predictor: knowledge						Predictor: beliefs of risk				
	I think that ChatGPT makes my job easier.	ChatGPT can be beneficial in health care settings.	ChatGPT provides trustworthy health care information or guidance.	ChatGPT is a useful tool when I need to search for information on specific medical questions.	ChatGPT is a useful tool when I need to search for medical literature.	I think that ChatGPT makes my job easier.		ChatGPT can be beneficial in health care settings.	ChatGPT provides trustworthy health care information or guidance.	ChatGPT is a useful tool when I need to search for information on specific medical questions.	ChatGPT is a useful tool when I need to search for medical literature.
Knowledge (1-5), OR^b^ (SE)	1.259^c^ (1.047-1.513)	1.468^d^ (1.289-1.672)	1.480^d^ (1.357-1.614)	1.448^d^ (1.400-1.498)	1.298^d^ (1.134-1.486)	—^e^		—	—	—	—
Beliefs of risk median (1-5), OR (SE)	—	—	—	—	—	2.040^d^ (1.765-2.358)		1.106^c^ (1.031-1.186)	1.062^f^ (1.013-1.113)	1.179^d^ (1.110-1.255)	1.138^d^ (1.076-1.203)
Age in years, OR (SE)	1.032^c^ (1.010-1.054)	1.036^d^ (1.014-1.058)	1.018 (0.993-1.044)	1.028^f^ (1.004-1.053)	1.043^d^ (1.024-1.063)	1.033^f^ (1.006-1.059)		1.049^d^ (1.029-1.069)	1.015^f^ (1.003-1.027)	1.042^d^ (1.025-1.060)	1.041^c^ (1.012-1.071)
Female (reference: male), OR (SE)	1.163^d^ (1.118-1.209)	0.712^d^ (0.696-0.729)	0.823^c^ (0.745-0.909)	0.863^f^ (0.782-0.953)	0.895 (0.783-1.024)	1.072 (0.977-1.176)		0.678^d^ (0.636-0.722)	0.822^c^ (0.710-0.950)	0.809 (0.651-1.005)	0.818^f^ (0.695-0.961)
Nonbinary or third gender (reference: male), OR (SE)	0.471^d^ (0.456-0.486)	1.662^d^ (1.548-1.784)	0.916 (0.843-0.995)	0.509^d^ (0.492-0.527)	0.921^f^ (0.865-0.981)	0.313^d^ (0.289-0.339)		2.648^d^ (2.552-2.748)	1.104 (0.997-1.224)	0.526^d^ (0.471-0.586)	1.900^d^ (1.800-2.006)
Prefer not to say (reference: male), OR (SE)	0.384^d^ (0.367-0.402)	0.438^d^ (0.424-0.452)	0.953 (0.876-1.036)	0.657^d^ (0.631-0.684)	1.291^d^ (1.150-1.450)	0.482^d^ (0.426-0.544)		0.461^d^ (0.439-0.485)	1.310^d^ (1.202-1.428)	0.557^d^ (0.508-0.611)	0.913^f^ (0.850-0.980)
Private institution (reference: public), OR (SE)	0.917 (0.791-1.063)	1.009 (0.917-1.111)	1.095 (0.911-1.316)	1.237^d^ (1.096-1.396)	1.348^d^ (1.104-1.646)	0.939 (0.768-1.148)		1.283^c^ (1.074-1.534)	1.206 (0.984-1.478)	1.608^d^ (1.328-1.946)	1.402^c^ (1.105-1.779)
Nursing (reference: medicine), OR (SE)	1.519^d^ (1.322-1.745)	0.956^f^ (0.921-0.992)	1.705^d^ (1.595-1.822)	1.867^d^ (1.784-1.954)	3.552^d^ (2.516-5.015)	1.973^d^ (1.855-2.098)		0.485^d^ (0.470-0.501)	0.559^d^ (0.543-0.575)	1.486^d^ (1.436-1.537)	3.909^d^ (3.525-4.336)
Nutrition (reference: medicine), OR (SE)	0.879^d^ (0.843-0.917)	0.437^d^ (0.432-0.442)	0.340^d^ (0.335-0.345)	0.503^d^ (0.495-0.511)	1.287^d^ (1.238-1.338)	0.676^d^ (0.629-0.726)		0.271^d^ (0.257-0.287)	0.175^d^ (0.168-0.182)	0.336^d^ (0.312-0.362)	1.401^d^ (1.264-1.553)
Dentistry (reference: medicine), OR (SE)	0.884 (0.785-0.996)	0.949^f^ (0.909-0.990)	1.275^d^ (1.210-1.344)	0.922^d^ (0.884-0.961)	1.113^d^ (1.052-1.178)	0.957 (0.810-1.131)		1.031 (0.939-1.133)	1.197^d^ (1.139-1.257)	0.923^f^ (0.863-0.987)	1.235^d^ (1.115-1.366)
Therapy (reference: medicine), OR (SE)	0.861 (0.470-1.578)	0.938 (0.878-1.002)	1.712^d^ (1.412-2.075)	1.551^d^ (1.054-2.282)	1.292^c^ (1.029-1.622)	1.020 (0.729-1.428)		0.868 (0.710-1.060)	1.776^f^ (1.032-3.056)	1.317^d^ (1.212-1.432)	0.948 (0.703-1.279)
Psychology (reference: medicine), OR (SE)	0.848 (0.666-1.079)	0.223^d^ (0.198-0.252)	0.428^c^ (0.338-0.541)	0.413^d^ (0.356-0.480)	0.642 (0.467-0.882)	0.675^f^ (0.477-0.955)		0.116^d^ (0.069-0.195)	0.450 (0.155-1.307)	0.325^d^ (0.176-0.599)	1.064 (0.778-1.456)
Pharmacology (reference: medicine), OR (SE)	0.0912^d^ (0.090-0.092)	1.946^d^ (1.692-2.238)	5.509^d^ (1.706-17.787)	2.603^d^ (1.974-3.432)	1.368^d^ (1.103-1.697)	0.236^d^ (0.198-0.281)		1.703^d^ (1.429-2.028)	4.976^d^ (4.039-6.135)	2.740^d^ (2.177-3.449)	1.506^d^ (1.324-1.711)
Other, OR (SE)	1.513^d^ (1.168-1.960)	1.073 (0.832-1.384)	1.667^d^ (1.428-1.946)	1.196^f^ (0.972-1.472)	1.261 (0.877-1.812)	1.307^f^ (1.005-1.699)		1.337 (0.790-2.261)	1.751^d^ (1.565-1.958)	1.166 (0.802-1.696)	1.329 (0.730-2.418)
/cut 1	0.245^d^ (0.223-0.269)	0.0945^d^ (0.091-0.098)	0.181^d^ (0.170-0.193)	0.142^d^ (0.136-0.149)	0.203^d^ (0.178-0.232)	0.966 (0.694-1.344)		0.0246^d^ (0.017-0.035)	0.0537^d^ (0.038-0.076)	0.0749^d^ (0.027-0.209)	0.100^d^ (0.040-0.254)
/cut 2	0.688 (0.527-0.898)	0.336^d^ (0.294-0.384)	0.764 (0.593-0.984)	0.479^d^ (0.408-0.562)	0.710 (0.469-1.076)	3.060^d^ (2.266-4.133)		0.151^d^ (0.116-0.198)	0.282^d^ (0.197-0.404)	0.370^c^ (0.194-0.705)	0.492 (0.221-1.095)
/cut 3	2.505^d^ (0.964-6.507)	1.704^c^ (0.879-3.305)	2.997^d^ (1.086-8.272)	1.845^c^ (0.936-3.635)	2.772^d^ (0.613-12.538)	12.66^d^ (9.459-16.945)		0.857 (0.657-1.119)	0.952 (0.727-1.245)	1.234 (0.733-2.077)	1.868 (0.873-3.995)
/cut 4	16.42^d^ (0.036-7565.397)	9.010^d^ (0.235-345.812)	19.50^d^ (0.044-8571.641)	11.36^d^ (0.221-583.882)	14.13^d^ (0.006-33,909.829)	96.46^d^ (72.024-129.153)		5.061^d^ (3.721-6.883)	6.823^d^ (5.038-9.235)	9.192^d^ (4.894-17.271)	9.435^d^ (4.865-18.302)

^a^Observations: predictor (knowledge): “I think that ChatGPT makes my job easier” n=863, “ChatGPT can be beneficial in health care settings” n=1513, “ChatGPT provides trustworthy health care information or guidance” n=1507, “ChatGPT is a useful tool when I need to search for information on specific medical questions” n=1501, and “ChatGPT is a useful tool when I need to search for medical literature” n=1490. Predictor (beliefs of risk): “I think that ChatGPT makes my job easier” n=861, “ChatGPT can be beneficial in health care settings” n=860, “ChatGPT provides trustworthy health care information or guidance” n=856, “ChatGPT is a useful tool when I need to search for information on specific medical questions” n=854, and “ChatGPT is a useful tool when I need to search for medical literature” n=849.

^b^OR: odds ratio.

^c^*P*<.01.

^d^*P*<.001.

^e^Not applicable.

^f^*P*<.05.

**Table 6:** Estimates from ordinal logistic regression models for the effect of perception scores on attitude variables (continuation)^a^.

Variables and outcomes	Predictor: ethics					Predictor: frequency of use				
	I think that ChatGPT makes my job easier.	ChatGPT can be beneficial in health care settings.	ChatGPT provides trustworthy health care information or guidance.	ChatGPT is a useful tool when I need to search for information on specific medical questions.	ChatGPT is a useful tool when I need to search for medical literature.	I think that ChatGPT makes my job easier.	ChatGPT can be beneficial in health care settings.	ChatGPT provides trustworthy health care information or guidance.	ChatGPT is a useful tool when I need to search for information on specific medical questions.	ChatGPT is a useful tool when I need to search for medical literature.
Ethics median (1-5), OR^b^ (IQR)	1.439^c^ (1.376-1.505)	1.495^c^ (1.452-1.539)	1.620^c^ (1.498-1.752)	1.476^c^ (1.430-1.523)	1.494^a^ (1.426-1.564)	—^d^	—	—	—	—
ChatGPT use frequency (1-5), OR (IQR)	—	—	—	—	—	1.320^c^ (1.199-1.454)	1.540^c^ (1.420-1.670)	1.365^c^ (1.321-1.410)	1.438^c^ (1.311-1.577)	1.396^c^ (1.302-1.497)
Age in years, OR (IQR)	1.030^e^ (1.011-1.049)	1.035^e^ (1.010-1.060)	1.015 (0.982-1.049)	1.029 (0.999-1.060)	1.043^c^ (1.021-1.065)	1.035^c^ (1.014-1.057)	1.061^c^ (1.036-1.087)	1.022 (0.993-1.051)	1.051^c^ (1.034-1.068)	1.046^c^ (1.022-1.071)
Female (reference: male), OR (IQR)	1.105^f^ (1.018-1.198)	0.682^c^ (0.648-0.718)	0.782^e^ (0.670-0.912)	0.827^f^ (0.711-0.961)	0.870 (0.742-1.019)	1.230^c^ (1.114-1.358)	0.797^c^ (0.768-0.827)	0.939 (0.816-1.080)	0.952 (0.752-1.204)	0.955 (0.805-1.132)
Nonbinary or third gender (reference: male)	0.599^c^ (0.568-0.631)	1.984^c^ (1.902-2.071)	1.223^c^ (1.155-1.294)	0.685^c^ (0.621-0.755)	1.110^e^ (1.041-1.183)	0.380^c^ (0.369-0.392)	2.174^c^ (1.780-2.655)	0.892 (0.791-1.005)	0.334^c^ (0.326-0.342)	1.583^c^ (1.406-1.782)
Prefer not to say (reference: male), OR (IQR)	0.499^c^ (0.438-0.567)	0.387^c^ (0.358-0.418)	0.822^c^ (0.739-0.913)	0.588^c^ (0.547-0.631)	1.141^e^ (1.036-1.257)	0.369^c^ (0.357-0.381)	0.469^c^ (0.454-0.484)	1.246^c^ (1.099-1.412)	0.492^c^ (0.473-0.511)	0.833^c^ (0.778-0.892)
Private institution (reference: public), OR (IQR)	0.951 (0.791-1.142)	1.077 (0.956-1.213)	1.197^c^ (1.091-1.313)	1.311^c^ (1.247-1.379)	1.438^c^ (1.261-1.639)	0.937 (0.803-1.094)	1.257^e^ (1.011-1.563)	1.181^f^ (0.974-1.432)	1.563^c^ (1.286-1.900)	1.403^c^ (1.087-1.810)
Nursing (reference: medicine), OR (IQR)	1.662^c^ (1.602-1.726)	1.006 (0.986-1.027)	1.713^c^ (1.589-1.846)	2.084^c^ (2.042-2.125)	3.732^c^ (3.504-3.971)	1.425^c^ (1.351-1.503)	0.350^c^ (0.340-0.360)	0.458^c^ (0.448-0.468)	1.237^c^ (1.192-1.284)	3.504^c^ (2.496-4.918)
Nutrition (reference: medicine), OR (IQR)	0.867^c^ (0.824-0.913)	0.392^c^ (0.376-0.408)	0.307^c^ (0.291-0.323)	0.459^c^ (0.445-0.472)	1.218^c^ (1.177-1.260)	0.901^c^ (0.858-0.947)	0.307^c^ (0.303-0.311)	0.187^c^ (0.185-0.189)	0.378^c^ (0.371-0.385)	1.545^c^ (1.354-1.763)
Dentistry (reference: medicine), OR (IQR)	0.838^f^ (0.712-0.987)	0.844^c^ (0.799-0.893)	1.152^c^ (1.095-1.213)	0.842^c^ (0.799-0.887)	1.028 (0.980-1.078)	0.992 (0.857-1.149)	1.258^c^ (1.088-1.455)	1.358^c^ (1.227-1.503)	1.036 (0.972-1.104)	1.412^c^ (1.261-1.581)
Therapy (reference: medicine), OR (IQR)	0.815 (0.536-1.240)	0.968 (0.757-1.239)	1.895^c^ (1.539-2.335)	1.552^c^ (1.467-1.642)	1.348 (0.960-1.893)	0.925 (0.582-1.469)	0.906 (0.715-1.148)	1.820^c^ (1.001-3.309)	1.292 (0.876-1.905)	0.947 (0.830-1.081)
Psychology (reference: medicine), OR (IQR)	0.819 (0.596-1.126)	0.197^c^ (0.112-0.345)	0.404^f^ (0.173-0.941)	0.397^c^ (0.287-0.548)	0.651 (0.325-1.303)	0.818 (0.628-1.066)	0.142^c^ (0.134-0.151)	0.519 (0.302-0.893)	0.375^c^ (0.306-0.459)	1.265 (0.924-1.731)
Pharmacology (reference: medicine), OR (IQR)	0.0920^c^ (0.084-0.101)	2.064^c^ (1.929-2.210)	6.336^c^ (5.501-7.294)	2.787^c^ (2.492-3.117)	1.545^c^ (1.397-1.709)	0.0774^c^ (0.077-0.078)	2.121^c^ (1.631-2.758)	4.864^c^ (1.748-13.531)	2.125^c^ (1.456-3.102)	1.196^e^ (1.018-1.405)
Other, OR (IQR)	1.394^c^ (1.221-1.590)	0.886 (0.707-1.111)	1.450^c^ (1.287-1.636)	1.012 (0.851-1.203)	1.085 (0.824-1.428)	1.493^c^ (1.291-1.727)	1.434 (0.772-2.664)	1.866^c^ (1.191-2.923)	1.276 (0.931-1.749)	1.432 (0.713-2.877)
/cut 1	0.280^c^ (0.187-0.418)	0.0852^c^ (0.047-0.153)	0.187^c^ (0.085-0.408)	0.134^c^ (0.091-0.198)	0.265^c^ (0.183-0.384)	0.259^c^ (0.228-0.294)	0.145^c^ (0.135-0.156)	0.115^c^ (0.110-0.120)	0.130^c^ (0.116-0.146)	0.178^c^ (0.151-0.210)
/cut 2	0.797 (0.559-1.137)	0.305^c^ (0.155-0.602)	0.807 (0.382-1.709)	0.457^c^ (0.288-0.725)	0.941 (0.642-1.379)	0.726 (0.528-0.997)	0.909 (0.653-1.266)	0.612^f^ (0.464-0.807)	0.645 (0.444-0.938)	0.881 (0.445-1.746)
/cut 3	2.980^c^ (2.090-4.250)	1.549 (0.817-2.939)	3.221^e^ (1.576-6.580)	1.761^f^ (1.027-3.019)	3.744^c^ (2.487-5.635)	2.677^c^ (0.815-8.797)	5.434^c^ (0.553-53.412)	2.119^c^ (0.839-5.355)	2.176^e^ (0.787-6.018)	3.425^e^ (0.254-46.156)
/cut 4	20.14^c^ (13.437-30.175)	8.187^c^ (4.554-14.717)	21.32^c^ (10.848-41.888)	10.79^c^ (6.527-17.832)	19.60^c^ (12.642-30.387)	18.23^c^ (0.002-164,263.740)	35.59^c^ (0.000-431,675,496.970)	15.94^c^ (0.040-6402.678)	17.24^c^ (0.001-441,561.358)	18.22^c^ (0.000-5,099,024.943)

^a^Observations: predictor (ethics): “I think that ChatGPT makes my job easier” n=863, “ChatGPT can be beneficial in health care settings” n=1513, “ChatGPT provides trustworthy health care information or guidance” n=1507, “ChatGPT is a useful tool when I need to search for information on specific medical questions” n=1501, and “ChatGPT is a useful tool when I need to search for medical literature” n=1490. Predictor (frequency of use): “I think that ChatGPT makes my job easier=863, ChatGPT can be beneficial in health care settings” n=861, “ChatGPT provides trustworthy health care information or guidance” n=860, “ChatGPT is a useful tool when I need to search for information on specific medical questions” n=858, and “ChatGPT is a useful tool when I need to search for medical literature” n=853.

^b^OR: odds ratio.

^c^*P*<.001.

^d^Not applicable.

^e^*P*<.01.

^f^*P*<.05.

## Discussion

### Principal Findings

The aim of this study was to determine the perception, attitudes, and uses of ChatGPT among health care students, as well as their willingness to learn more about it. Given that chatbots powered by AI are widely accepted by students [27], our findings provide critical insights into the possibilities of integrating them into undergraduate health care teaching programs. More than half (1419/2661, 53.32%) of the participants knew about ChatGPT according to our data, with male students being more knowledgeable than female students. In May 2023, the Pew Research Center released the findings of a web-based study that showed that, compared to our results (1142/2661, 42.92%), 33% of young people had never heard of ChatGPT. Most participants felt that they knew little to nothing about ChatGPT [28]. According to the study by Buabbas et al [29], 84% of Kuwaiti medical students did not have any training on the use of AI. It is worth noting that >80% of our participants (2160/2661, 81.17%) indicated an interest in learning more about ChatGPT’s health care applications, with time restrictions being the primary barrier to learning more for 39.98% (1064/2661) of them.

Despite the widespread use of AI chatbots such as ChatGPT for self-diagnosing illnesses (up to 78%) [30] and the recognition of the value and user-friendliness of the information they provide, health care career students in the Americas maintained a neutral stance on whether ChatGPT will replace their jobs. They neither agreed nor disagreed with the notion. This aligns with the findings of the studies by Buabbas et al [29] and Moldt et al [31], where 78.7% and 83% of participants, respectively, expressed skepticism about AI eventually replacing the roles of physicians in the future.

Only 22.98% (349/1519) of our students reported using AI for medical and health care education and training, but >70% (1101/1519, 72.48%) said that they used it for homework support. Although some colleges prohibit the use of ChatGPT and consider it plagiarism [32], teachers are investigating its utility during learning. For example, the students of Mullen [33] used ChatGPT to improve the quality of an essay in English (their nonnative language), and the participants felt that the experience left them better equipped to produce future academic output without the use of these tools.

Our study revealed that health care students displayed positive attitudes and acceptance toward ChatGPT and that most were willing to learn more about it, similar to the studies by Buabbas et al [29] and Moldt et al [31]. Although we did not inquire about the specific version of ChatGPT used by participants, and as ChatGPT’s primary function is not to be used as a web search engine, it is evident that, within the context of higher education, particularly in the field of health, there has been a significant increase in the adoption of disruptive technologies [34], including ChatGPT, as both formal or informal tools for enhancing skills and achieving educational objectives [35].

Respondents perceived ChatGPT as a valuable tool in health care settings, highlighting its usefulness in providing information on specific medical questions and facilitating access to relevant literature. Interestingly, the attitudes toward ChatGPT appeared to be influenced by the participants’ self-perceived knowledge about the chatbot. Those who had a better understanding of ChatGPT tended to perceive it as providing trustworthy health care information or guidance. Notably, participants’ willingness to use ChatGPT in the health care setting is heavily influenced by the level of trust they have in the system [6]. Interestingly, we found a significant association between increased perceived risk scores and the following attitude statement: “ChatGPT provides trustworthy health care information or guidance.” Establishing trust is crucial to ensuring the responsible and effective use of ChatGPT, thereby maximizing its benefits while mitigating any associated risks.

Indeed, this study revealed that users’ attitudes toward ChatGPT are positively influenced by the frequency of use. Individuals who use ChatGPT more frequently have higher possibilities of believing that ChatGPT makes their job easier and finding it beneficial in health care settings, as well as considering it a useful tool for searching specific medical questions and medical literature. Despite students being somewhat concerned about the perceived risk of the ethical implications of using ChatGPT, they still used it once a month, especially for homework support, research paper writing support, medical or health care education and training, and mental health support. Our study differs from previous research, and Firaina and Sulisworo [36] found that most respondents preferred frequent use of ChatGPT.

Despite the many changes that have occurred in medicine over the last few decades, medical education is still largely based on traditional teaching methods [37,38]. The release of ChatGPT caused concerns and debates in health care due to ethical issues, misinformation, misuse, and challenges in practice and academic writing. Concerns include the quality and dependability of medical information, the chatbot model’s transparency, the ethics of user information, and potential biases in the ChatGPT algorithms [35]. While several studies have demonstrated ChatGPT’s ability to answer medical questions [39-42], many correct answers have been deemed inadequate [39,40].

### Limitations

Our study has several limitations that must be considered when interpreting the results. First, our sampling strategy did not capture all health care students from the Americas. Despite our efforts to include universities across the Americas, we encountered a limited recruitment response from Central America. This low number may limit the representativeness of our findings for this specific region. As a result, the findings from Central America should be considered as preliminary and require validation through larger-scale research conducted in this region. Second, this study was cross-sectional in nature, and, therefore, we cannot establish causality among perceptions, beliefs, ethics, and attitudes. Longitudinal studies are needed to determine the temporal relationship between these variables. Third, although, during the course of this study, there were 2 available versions of ChatGPT (3.5 and 4.0), the participants were not specifically queried on which version they used. However, given their status as students, it can be reasonably deduced that they predominantly used the free version rather than the premium version. The disparities between the 2 versions lie mostly in the payment requirement associated with version 4.0. It has been said that this particular version offers enhanced safety measures, more valuable responses, and a heightened comprehension of the contextual nuances pertaining to the posed queries. On the basis of the aforementioned findings, certain worries emerge regarding the potential use of ChatGPT by students within their educational institutions but in an informal manner despite the absence of official integration of ChatGPT as an explicitly disruptive technological tool within their educational system. It is also possible that academic institutions are incorporating this technology within their instructional settings. At present, there remain unanswered inquiries pertaining to the subject matter. However, these discoveries indicate potential gaps in knowledge, warranting an assessment of whether the acquired information satisfies the minimum criteria for quality in the field of health and possesses genuine value in terms of gathering competent professionals in the near future.

### Conclusions

The current debate revolves around the potential advantages and disadvantages of incorporating ChatGPT and other LLMs into the teaching and learning process. The age of AI has arrived. It is important to be aware of how it may be used and misused. Research in health care education looks bright in the future due to the essential integrity that drives the vast majority of researchers. A medical educator must remain current with the rapid advancements in technology and consider how they affect their teaching practices, curriculum development, and evaluation techniques.

## Appendix

Multimedia Appendix 1 Original version of the survey administered to the participants during the study.

Multimedia Appendix 2 Detailed breakdown of the demographic characteristics of a sample population consisting of 2661 individuals. The data include the composition of the sample population in terms of age, gender, type of university, geographic region, and academic majors.

Multimedia Appendix 3 Distribution of ChatGPT use by health care students across various academic and clinical activities, indicating the range of use and the percentage of students who use ChatGPT for each activity.

## Abbreviations

AI: artificial intelligence
LLM: large language model
OR: odds ratio

## Data Availability

All data generated or analyzed during this study are included in this published article and its supplementary information files.
