# Supplementary material for: Understanding Health Care Students’ Perceptions, Beliefs, and Attitudes Toward AI-Powered Language Models: Cross-Sectional Study
Source: JMIR Med Educ. 2024 Aug 13;10:e51757. doi: 10.2196/51757 (PMC11350293; doi:10.2196/51757)
Supplement: Multimedia Appendix 1 [file mededu_v10i1e51757_app1.docx]

Start of Block: Default Question Block

Q1 **Title**: Knowledge, Attitudes, Beliefs, and Practice Regarding ChatGPT Among Healthcare College Students
 **Objective**: To assess the knowledge, attitudes, beliefs, and practices of healthcare professionals concerning ChatGPT, an AI-powered language model, and its potential use in clinical settings.
 **Informed Consent Statement**: By clicking "I accept" ; you confirm that you voluntarily agree to participate in this research study. Your participation will involve answering a series of questions related to ChatGPT, its applications in healthcare settings, and your personal beliefs and practices. Your responses will be kept confidential and will only be used for research purposes.

- I accept (1)
- I don't accept (2)

Skip To: End of Survey If Q1 = 2

End of Block: Default Question Block

Start of Block: Block 1

| 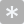 |
| --- |

Q2 What is your age? (in years) Insert only the number.

________________________________________________________________

Q3 What is your current gender?

- Male (1)
- Female (2)
- Non-binary / third gender (3)
- Prefer not to say (4)
- Other (5) __________________________________________________

Q4 Your college/university is:

- Public (1)
- Private (2)
- Both (3)

Q27 In which country is the university where you study located?

________________________________________________________________

| Page Break |  |
| --- | --- |

Q5 What major do you study? (Select the option that best applies to you)

- Medicine (1)
- Nursing (2)
- Nutrition (3)
- Dentistry (4)
- Therapist (if you choose this write the type of therapy, for example, Physical) (5) __________________________________________________
- Psychologist (6)
- Pharmacologist (7)
- Other: (8) __________________________________________________

| 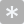 |
| --- |

Q7 How many years have you been studying this major?

________________________________________________________________

End of Block: Block 1

Start of Block: Block Loop

Q8 Have you heard of ChatGPT before?

- Yes (1)
- No (2)

End of Block: Block Loop

Start of Block: Block 2

Display This Question:

If Q8 = 1

Q9 How would you rate your knowledge of ChatGPT and its applications in healthcare?

- No knowledge (1)
- Minimal knowledge (2)
- Basic knowledge (3)
- Adequate knowledge (4)
- Superior knowledge (5)

Display This Question:

If Q8 = 1

Q10 Which of the following applications of ChatGPT in healthcare are you aware of? (select all that apply)

- **Electronic health record documentation** (managing patient information digitally) (1)
- **Patient triage** (assessing and prioritizing patients based on the severity of their condition) (2)
- **Medical/Healthcare education and training** (learning or teaching medical knowledge and skills) (3)
- **Clinical decision support** (tools or systems to assist healthcare providers in making informed decisions) (4)
- **Mental health support** (counseling, therapy, or resources for emotional well-being) (5)
- **Health communication** (effective exchange of health-related information among patients, providers, and organizations) (6)
- **Research writing support** (assistance with writing and editing research papers, grant proposals, or theses) (7)
- **Homework support** (help with understanding and completing medical or healthcare-related assignments) (8)

End of Block: Block 2

Start of Block: Block 3

Display This Question:

If Q8 = 1

Q11 Please indicate your level of agreement with the following statements:

|  | Strongly agree (1) | Somewhat agree (2) | Neither agree nor disagree (3) | Somewhat disagree (4) | Strongly disagree (5) |
| --- | --- | --- | --- | --- | --- |
| "I believe that AI-powered language models like ChatGPT can be beneficial in healthcare settings." (1) |  |  |  |  |  |
| "I trust AI-powered language models like ChatGPT to provide trustworthy healthcare information or guidance." (2) |  |  |  |  |  |
| "I believe that AI-powered language models like ChatGPT are useful tools when I need to search for information on specific medical questions." (3) |  |  |  |  |  |
| "I believe that AI-powered language models like ChatGPT are useful tools when I need to search for medical literature". (4) |  |  |  |  |  |

Display This Question:

If Q8 = 1

Q15 What concerns do you have regarding the use of AI-powered language models like ChatGPT in healthcare? (select all that apply)

- Patient privacy and data security (1)
- Misdiagnosis or incorrect information (2)
- Over-reliance on AI, leading to decreased clinical skills (3)
- Potential bias in AI algorithms (4)
- Loss of human touch and empathy in patient care (5)
- Legal and ethical implications (6)
- None (7)
- Other (8) __________________________________________________

Display This Question:

If Q8 = 1

Q17 How ethical do you consider the use of ChatGPT-like tools for the following scenarios:

|  | Totally unethical (1) | Somewhat unethical (2) | Neither ethical nor unethical (3) | Somewhat ethical (4) | Totally ethical (5) |
| --- | --- | --- | --- | --- | --- |
| Revising the language of scientific manuscript? (1) |  |  |  |  |  |
| Writing text in a scientific manuscript? (2) |  |  |  |  |  |
| The sole source of information for clinical practice? (3) |  |  |  |  |  |

End of Block: Block 3

Start of Block: Block 4

Display This Question:

If Q8 = 1

Q16 Have you ever used ChatGPT before?

- No (1)
- Yes (2)

Display This Question:

If Q8 = 1

And Q16 = 2

Q18 Do you use ChatGPT or any similar AI-powered language models in your daily life?

- No (1)
- Yes (2)

Display This Question:

If Q8 = 1

And Q16 = 2

Q19 If you have used ChatGPT or similar tools, which of the following applications have you used it for? (select all that apply)

- **Electronic health record documentation** (managing patient information digitally) (1)
- **Patient triage** (assessing and prioritizing patients based on the severity of their condition) (2)
- **Medical/Healthcare education and training** (learning or teaching medical knowledge and skills) (3)
- **Clinical decision support** (tools or systems to assist healthcare providers in making informed decisions) (4)
- **Mental health support** (counseling, therapy, or resources for emotional well-being) (5)
- **Health communication** (effective exchange of health-related information among patients, providers, and organizations) (6)
- **Research writing support** (assistance with writing and editing research papers, grant proposals, or theses) (7)
- **Homework support** (help with understanding and completing medical or healthcare-related assignments) (8)

Display This Question:

If Q8 = 1

And Q16 = 2

Q20 How frequently do you use ChatGPT now?

- More than once a day (1)
- Once a day (2)
- Once a week (3)
- Once a month (4)
- Less than once a month (5)

Display This Question:

If Q8 = 1

And Q16 = 2

Q21 How frequently do you anticipate using ChatGPT six months from now?

- More than once a day (1)
- Once a day (2)
- Once a week (3)
- Once a month (4)
- Less than once a month (5)

End of Block: Block 4

Start of Block: Block 5

Display This Question:

If Q8 = 1

And Q16 = 2

Q22 Please indicate your level of agreement with the following statements (rate on a scale from Strongly disagree to Strongly agree):

|  | Strongly disagree (1) | Somewhat disagree (2) | Neither agree nor disagree (3) | Somewhat agree (4) | Strongly agree (5) |
| --- | --- | --- | --- | --- | --- |
| "I think that ChatGPT makes my job easier." (1) |  |  |  |  |  |
| "I think my job could be replaced in the future because of AI." (2) |  |  |  |  |  |
| "I believe that, in the future, ChatGPT (or some similar technology) will play an ever more important role in my job". (3) |  |  |  |  |  |
| "I believe that using AI like ChatGPT in clinical practice raises ethical concerns." (4) |  |  |  |  |  |

End of Block: Block 5

Start of Block: Block 6

Q23 Would you like to learn more about ChatGPT and its potential applications in healthcare?

- Yes (1)
- No (2)

Display This Question:

If Q23 = 1

Q24 Which aspects of ChatGPT and its applications in healthcare are you most interested in learning about? (select all that apply)

- Specific use cases in medical practice (1)
- Integration with existing healthcare systems (2)
- Data privacy and security measures (3)
- Ethical considerations (4)
- Potential benefits and limitations (5)
- Academic homework support (6)
- Other (7) __________________________________________________

Display This Question:

If Q23 = 1

Q26 What resources or educational materials would you find most helpful in learning about ChatGPT and its potential applications in healthcare? (select all that apply)

- Research articles and case studies (1)
- Webinars or online courses (2)
- Workshops or conferences (3)
- Interactive demos or hands-on experience (4)
- Peer recommendations and testimonials (5)
- I don't know (6)
- Other (7) __________________________________________________

Display This Question:

If Q23 = 2

Q25 What are the reasons for your lack of interest in learning more about ChatGPT and its potential applications in healthcare? (select all that apply)

- Lack of time (1)
- Already overwhelmed with existing medical knowledge and skills (2)
- Lack of relevance to my medical specialty (3)
- Skepticism about the benefits of AI in healthcare (4)
- Not enough knowledge of these technologies (5)
- Difficulty or discomfort using computer technology (6)
- I prefer to consult with my peers, mentors and teachers (7)
- Other (8) __________________________________________________

Display This Question:

If Q23 = 1

Q27 Would you be interested in receiving more information about ChatGPT and its applications in healthcare?

- Yes (1)
- No (2)

Q28 Would you be willing to participate in a follow-up survey in a couple of months to assess if knowledge and attitudes towards ChatGPT have changed?

- Yes (1)
- No (2)
