# Supplementary material for: Understanding Health Care Students’ Perceptions, Beliefs, and Attitudes Toward AI-Powered Language Models: Cross-Sectional Study
Source: JMIR Med Educ. 2024 Aug 13;10:e51757. doi: 10.2196/51757 (PMC11350293; doi:10.2196/51757)
Supplement: Multimedia Appendix 2 [file mededu_v10i1e51757_app2.docx]

**Multimedia Appendix 2. Demographic information (n=2661)**

| **Variables** | **Total, % (n)** |
| --- | --- |
| **Age** | 21.65 |
| **Gender** |  |
| Male | 32.88% (875) |
| Female | 66.33% (1765) |
| Non-binary / third gender | 0.30% (8) |
| Prefer not to say | 0.38% (10) |
| Other | 0.11% (3) |
| **Type of University** |  |
| Public | 30.97% (824) |
| Private | 69.03% (1837) |
| **Region** |  |
| Central America | 8.19% (218) |
| South America | 91.81% (2443) |
| **Major** |  |
| Medicine | 33.41% (889) |
| Nursing | 1.84% (49) |
| Nutrition | 2.18% (58) |
| Dentistry | 55.09% (1466) |
| Therapist | 1.65% (44) |
| Psychologist | 1.69% (45) |
| Pharmacologist | 0.56% (15) |
| Other | 3.57% (95) |

Note: For numerical data, results are displayed as "Mean (Standard Deviation)". For categorical data, they are presented as "Percentage (Count)".
