# Supplementary material for: Understanding Health Care Students’ Perceptions, Beliefs, and Attitudes Toward AI-Powered Language Models: Cross-Sectional Study
Source: JMIR Med Educ. 2024 Aug 13;10:e51757. doi: 10.2196/51757 (PMC11350293; doi:10.2196/51757)
Supplement: Multimedia Appendix 3 [file mededu_v10i1e51757_app3.docx]

Multimedia Appendix 3. Distribution of ChatGPT Utilization Among Healthcare Students

| Item | | N, (%) |
| --- | --- | --- |
|  | Electronic health record documentation | 167/1518 (11.00) |
|  | Patient triage | 106/1514 (7.00) |
|  | Medical or healthcare education and training | 349/1517 (23.00) |
|  | Clinical decision support | 122/1525 (8.00) |
|  | Mental health support | 213/1521 (14.00) |
|  | Health communication | 167/1518 (11.00) |
|  | Research writing support | 637/1517 (42.00) |
|  | Homework support | 1078/1518 (71.00) |
